# DMT1 Expression and Iron Levels at the Crossroads Between Aging and Neurodegeneration

**DOI:** 10.3389/fnins.2019.00575

**Published:** 2019-06-05

**Authors:** Rosaria Ingrassia, Barbara Garavaglia, Maurizio Memo

**Affiliations:** ^1^Section of Biotechnologies, Department of Molecular and Translational Medicine, University of Brescia, Brescia, Italy; ^2^Medical Genetics and Neurogenetics Unit, Fondazione IRCCS Istituto Neurologico Carlo Besta, Milan, Italy; ^3^Section of Pharmacology, Department of Molecular and Translational Medicine, University of Brescia, Brescia, Italy

**Keywords:** iron homoeostasis, neurodegenerative diseases, aging, transport of iron and heavy metals, divalent metal transporter 1 up-regulation, non-transferrin bound iron transport, neurodegeneration with brain iron accumulation

## Abstract

Iron homeostasis is an essential prerequisite for metabolic and neurological functions throughout the healthy human life, with a dynamic interplay between intracellular and systemic iron metabolism. The development of different neurodegenerative diseases is associated with alterations of the intracellular transport of iron and heavy metals, principally mediated by Divalent Metal Transporter 1 (DMT1), responsible for Non-Transferrin Bound Iron transport (NTBI). In addition, DMT1 regulation and its compartmentalization in specific brain regions play important roles during aging. This review highlights the contribution of DMT1 to the physiological exchange and distribution of body iron and heavy metals during aging and neurodegenerative diseases. DMT1 also mediates the crosstalk between central nervous system and peripheral tissues, by systemic diffusion through the Blood Brain Barrier (BBB), with the involvement of peripheral iron homeostasis in association with inflammation. In conclusion, a survey about the role of DMT1 and iron will illustrate the complex panel of interrelationship with aging, neurodegeneration and neuroinflammation.

## Introduction

Interest in the contribution of iron and DMT1 to both aging and neurodegeneration comes from relevant studies performed during the course of the last 20 years. As Knutson (2017) importantly highlighted, last year was the 20th anniversary of the discovery of DMT1, the first reported mammalian divalent metal transporter. DMT1 principally mediates the transport of ferrous iron and heavy metals in systemic iron homeostasis, from the plasma membrane or endosomes to the intracellular labile pool, mostly sustaining Non-Transferrin Bound Iron transport (NTBI). Although according to recent highlights, the uptake of NTBI into the liver seems to be mediated by ZIP14/SLC39A14 (Jenkitkasemwong et al., 2012) and hepatocyte DMT1 was shown to be dispensable for hepatic iron accumulation (Wang and Knutson, 2013). Interestingly, ^59^Fe-NTBI uptake was impaired in ZIP14/SLC39A14 null mice when fed with iron-loaded diet or crossed with mouse models of hereditary hemochromatosis, while DMT1 siRNA reduced iron loading in a mouse model of hereditary hemochromatosis (Wang et al., 2019), thus highlighting the role of both NTBI transporters in the complex homeostasis of iron overload, with cerebral implications. Noteworthy, the comparison of these two NTBI-transporters activity, in RNA injected Xenopus oocytes, showed a maximum at pH 7.5 for mouse ZIP14, compared to human DMT1 (Pinilla-Tenas et al., 2011), a proton co-transporter, shows an optimum ferrous iron uptake at pH 5.5 (Garrick et al., 2006; Mackenzie et al., 2007), which could exacerbate iron uptake during the pathological-associated extracellular acidosis. Other non-transferrin-bound iron transporters were later identified, such as ZIP14 (SLC39A14), ZIP8 (SLC39A8), L-type calcium channels (LTCCs) and T-type calcium channels (TTCCs), and TRPC6 (Martinez et al., 1999; Mwanjewe and Grover, 2004; He et al., 2006; Liuzzi et al., 2006; Jenkitkasemwong et al., 2012; Striessnig et al., 2014). However, here attention will be given into the complex structure and function of DMT1, underpinning the equilibrium of iron and heavy metals homeostasis, as an essential prerequisite for normal metabolic and neurological functions in relationship to heterogeneous pathophysiological regulation. In this regard, iron and DMT1 significantly contribute to the development of different neurodegenerative diseases, assuming the role of a common participant, as later described. In this review, we discuss the growing body of findings and the emerging concepts about DMT1 and NTBI involvement in both physiological aging and neurodegeneration/neuroinflammation, as found in Parkinson’s disease (PD), ischemia, Neurodegeneration with Brain Iron Accumulation (NBIA), and Alzheimer’s disease (AD). To this purpose, focus will be given to the potential role of DMT1 as an interesting target for the development of future neuroprotective strategies in the field.

## Ferrous Iron Transporter DMT1 and Redox Equilibrium in the Life-Sustaining Cellular Homeostasis

**FIGURE 1:**
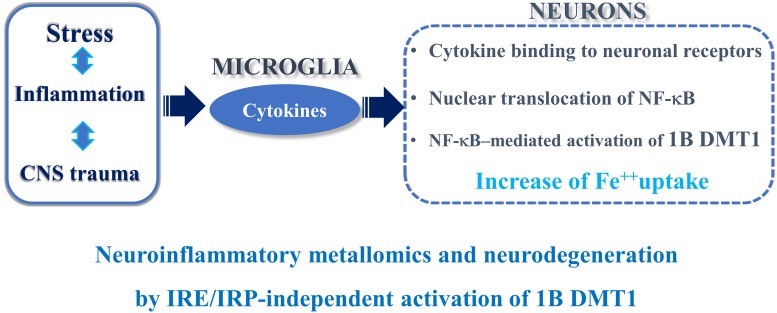
Neuroinflammation and IRE/IRP-independent activation of 1B DMT1.

Divalent Metal Transporter 1 (or Nramp2 gene) was first isolated by low stringency Nramp1 homology screening (Gruenheid et al., 1995) and expression cloning of a rat duodenal cDNA library (Gunshin et al., 1997). Subsequently, Lee et al. (1998) firstly defined the structure of the 3′UTR splice variant without-IRE and Kishi and Tabuchi (1997) performed functional characterization in yeast of human DMT1 cDNA (Tabuchi et al., 1999). DMT1 expression and function are strictly dependent on this complex structure, with four different isoforms, generated by two alternative splicing (Garrick et al., 2006; Mackenzie et al., 2007). The 5′UTR splicing produces two different promoter regions, 1A and 1B. The 1A promoter is responsive to hypoxia in PC12 rat cells (Lis et al., 2005) and HIF-2 alpha in Caco-2/TC7 cells (Mastrogiannaki et al., 2009). Conversely, the 1B isoform is responsive to NF-κB in P19 mouse embryonic carcinoma cells, mouse primary cortical neurons (Paradkar and Roth, 2006a, 2006b; Ingrassia et al., 2012) and to HIF-1 alpha in HepG2 cells (Wang et al., 2010; Qian et al., 2011). The second splicing, at 3′UTR, implies that either 1A and 1B isoforms possess or do not possess an Iron Responsive Element (IRE), that is sensitive to feedback regulation by intracellular iron levels, through IRE/IRP system (Hubert and Hentze, 2002; Pantopoulos, 2004). Moreover, in Caco-2 cell line, both 1A isoforms and (+) IRE 1B isoform show the predicted mRNAs up- and down-regulation in iron deficiency or overload, respectively, while the (–) IRE 1B isoform is regulated by iron-independent mechanisms (Hubert and Hentze, 2002). Particularly, Hubert and Hentze showed that, in Caco-2 cell line, the Transferrin Receptor (TfR) and (+) IRE DMT1 isoform are down-regulated by intracellular iron overload, due to the canonical IRE/IRP post-transcriptional control. In this respect, while (+) IRE DMT1 isoforms do not contribute to the increased uptake of ferrous iron during iron overload, (–) IRE DMT1 isoforms are not influenced by intracellular iron perturbation. Conversely, (–) IRE DMT1 can be regulated at the transcriptional level (Paradkar and Roth, 2006a, 2006b; Ingrassia et al., 2012; Figure 1), post-translationally, via impairment of proteasome degradation (Garrick et al., 2012; Jia et al., 2014) and by autophagy (Ingrassia et al., 2017). In this regard, IRP1, IRP2, (+) IRE and (–) IRE DMT1 proteins are up-regulated in lesioned rat hippocampus after kainate treatment and expressed in GFAP positive astrocytes (Huang et al., 2006); the (–) IRE DMT1 increased expression leads to argue that mechanisms other than IRP regulation modulate DMT1 expression.

Importantly, (–) IRE DMT1, which co-localizes with TfR on early endosomes, is also involved in the life-sustaining Transferrin Receptor cycle (Tabuchi et al., 2002, 2010), where the release of ferric iron from transferrin is triggered by endosomal acidic pH, followed by its reduction to ferrous iron with loading by DMT1, whose activity is maximally stimulated at low pH (Garrick et al., 2006; Mackenzie et al., 2007).

Divalent Metal Transporter 1 isoforms play multiple roles and are characterized by complex subcellular distribution; in fact, they localize at the cell membrane, in the cytoplasm and at the nuclear and outer mitochondrial membranes (Roth et al., 2000; Lis et al., 2004; Wolff et al., 2014a, 2014b), where they were recently found to play a role in mitochondrial iron and manganese uptake (Wolff et al., 2018). Through this heterogenous distribution, a tight control of ferrous iron homeostasis is guaranteed at intracellular level, avoiding, for example, excess of ferrous iron that may catalyze the formation of free radicals through the Haber–Weiss and Fenton reactions, and lead to cell damage (Connor and Ghio, 2009). In this context, pharmacological manipulation of the Fenton reaction, by preventing cellular oxidative damage and the formation of hydrogen peroxide and hydroxyl radicals, could represent a useful targeting system leading to potential therapeutic strategies in the treatment of neurodegenerative disorders (Youdim, 2018). Moreover, a physiological pathway for NTBI uptake and regulation of systemic iron homeostasis in different tissues has been extensively described (Knutson, 2018). In this scenario, DMT1 supports a crosstalk between peripheral districts and the central nervous system, with iron transport into the blood stream at the Blood Brain Barrier. According to previous results, DMT1 gene expression in the mouse brain presumably is mostly accounted for by 1B isoform, with both (+)/(–) IRE splicing, which has also been reported to be expressed in rat brain. Whereas no evidence for expression of 1A isoform was found (Hubert and Hentze, 2002; Ke et al., 2005). DMT1 mRNA was also found up-regulated in the cerebellum of ceruloplasmin-knockout mice, particularly in Purkinje and deep nuclei neurons (Jeong and David, 2006), in the frontal cortex of both wild-type and APP_SWE_/PS1_ΔE9_ Alzheimer mouse model (Xian-hui et al., 2015), and (+)/(–) IRE DMT1 isoforms significantly increased in rat cortex, striatum, hippocampus and substantia nigra (Lu et al., 2017). DMT1 is also controversially reported to be expressed not only in neurons, but also in non-neuronal cells, such as astrocytes, microglia, oligodendrocytes, and over the brain capillary endothelial cells and choroid plexus cells, that form the brain barrier (Moos and Morgan, 2004; Mills et al., 2010; Skjørringe et al., 2015). Indeed, rat brain capillaries *in vivo* and isolated endothelial cells, with Blood Brain Barrier (BBB) competence, express DMT1, transferrin receptor, ferroportin, ceruloplasmin, ferrireductases STEAP2-3, and hephestin. This complex machinery sustains the distribution of iron in the central nervous system with epithelial cells of the choroid plexus expressing large amounts of DMT1 mRNA, in order to regulate metal transport across the BBB (Yang W.M. et al., 2011; Burkhart et al., 2016). Divalent metal transport was also studied in cerebral hemorrhage models subjected to iron chelation by deferoxamine (Li et al., 2017), in induced pluripotent stem cell (iPSC)-derived brain endothelial cells (huECs) and a human BBB cellular model where transferrin, hepcidin, and DMT1 sustain iron transport and release (Chiou et al., 2018).

## DMT1 and Iron Up-Regulation During Aging

Several studies demonstrated how (–)/(+)IRE DMT1 mRNAs expression levels are significantly increased during aging in rat brain cortex, hippocampus, striatum and substantia nigra (Ke et al., 2005; Lu et al., 2017). The authors of these studies showed how (–)/(+)IRE DMT1 mRNAs have a peculiar compartmentalization in both early development and aging, with significant up-regulation at 3 post-natal weeks in the cortex and at 28 post-natal weeks in the substantia nigra, that is surprisingly not influenced by dietary intake in rats, in spite of the expected iron-dependent response. DMT1 mRNA was then found up-regulated in the cerebellum of 24-month-old ceruloplasmin-knockout mice, and DMT1 protein was found increased in Purkinje and deep nuclei neurons (Jeong and David, 2006). DMT1 mRNA was also higher in the frontal cortex of 12-month-old wild-type mice and, with significant increase, in the APP_SWE_/PS1_ΔE9_ Alzheimer mouse model (Xian-hui et al., 2015). However, (+)/(–) IRE DMT1 isoforms were found significantly up-regulated at the protein level in the cortex, striatum, hippocampus and substantia nigra of 3-, 12-, and 24-month-old rats, where a significant increase of hepcidin was also described by immunofluorescence (Lu et al., 2017), potentially sustaining a block of iron export. Interestingly, DMT1 up-regulation in the substantia nigra during aging links the transporter to the pathogenesis of other neurodegenerative diseases, such as PD, Parkinsonisms and NBIA, in which enhanced iron accumulation into the basal ganglia occurs. In this regard, in an NBIA mouse model with a mutation in phospholipase A2 beta (PLA2G6), aging significantly up-regulates IRPs and (+) IRE DMT1 in the cortex, striatum, substantia nigra and cerebellum of 100-weeks-old mice, with respect to age-matched wild-type controls (Beck et al., 2015). Furthermore, beside DMT1 increase during aging in the central nervous system, which is highly subjected to damage by iron-dependent oxidative stress, hepatic DMT1 protein was also found up-regulated by Deferoxamine treatment in 24-month-old rats, when compared to 3-month-old rats (Bloomer et al., 2014).

## DMT1 and Iron Up-Regulation in the Neurodegeneration

Oxidative stress leads to DNA damage and to the polymerization and denaturation of proteins that, together, can form insoluble structures typically known as plaques, a hallmark of neurodegenerative diseases (Kell, 2009), with altered cellular proteostasis. Arising from the previous reasons, the intracellular iron level and traffic need a tight control to avoid perturbations in the expression of DMT1, which may induce downstream cellular damage and dysfunctions in iron metabolism, contributing to the pathogenesis of several neurodegenerative disorders (Biasiotto et al., 2016). In this respect, DMT1 mRNA localization in rat central nervous system by *in situ* hybridization showed a restricted pattern throughout different areas, as reported by Gunshin et al. (1997), with prominent DMT1 labeling in cerebellar granule cells, hippocampal pyramidal and granule cells in the preoptic nucleus and pyramidal cells of the piriform cortex, as well as in the substantia nigra. The authors also detected DMT1 labeling in the ventral area of the anterior olfactory bulb and in the olfactory epithelium, an important route for the delivery of environmental metals to the brain. The intranasal drug delivery may exert highly efficient effects in the central nervous system (Henkin, 2011) and at the circulatory level, inducing an effective systemic immunity (McGhee, 2011). In fact, the olfactory epithelium can be considered as a gateway for vaccines or peptide hormones administration, also in light of the high neuroprotective potential showed by intranasal administration of iron chelators (Guo et al., 2015). Accordingly, intranasal administration of neurotoxins in rodents, such as 1-methyl-4-phenyl-1,2,3,6-tetrahydropyridine (MPTP), has been used to develop an animal model suitable for testing neuroprotective drugs against PD (Prediger et al., 2011).

An important aspect concerns the role of DMT1 in the pathogenesis of several neurodegenerative diseases induced by known neurotoxic stimuli such as kainate, N-methyl-D-aspartate (NMDA), agonist of the NMDA Receptors of the family of ionotropic glutamate receptors, levodopa or L-3,4-dihydroxyphenylalanine (L-DOPA), 1-methyl-4-phenyl-1,2,3,6-tetrahydropyridine (MPTP), 6-hydroxydopamine (6-OHDA) and hypoxia, as discussed later. DMT1 also plays a role in neuronal cell death in the hippocampus by excitotoxic glutamatergic stimuli, such as kainate and NMDA. DMT1, in fact, was shown to be up-regulated by kainate in rat hippocampus, both by an IRP-dependent and -independent mechanism, being the (+)IRE and the (–)IRE isoforms up-regulated and mostly expressed in GFAP positive astrocytes (Huang et al., 2006). The kainate-dependent DMT1 up-regulation and hippocampal injury were then associated with higher cerebral levels of lead and cadmium in rats, when treated with these metals dissolved in the drinking water (Ong et al., 2006). Interestingly, other reports suggested a possible role of DMT1 in the hippocampal spatial memory formation, due to the evidence of NMDA-dependent plasticity induction by 1B/(+)IRE DMT1 in rat primary hippocampal neurons, and evidence of its block by the transcription inhibitor Actinomycin D and the NMDA receptor antagonist MK-801 (Haeger et al., 2010). Thus, NMDA receptor can influence neurodegenerative diseases through DMT1 enhancement, with consequent iron overload-dependent NMDA-Receptor mediated synaptic plasticity. The NMDARs-dependent iron overload also challenges increased lysosomal iron release. In this regard, it was well reported that (–)/(+)IRE DMT1 isoforms have different subcellular localization (Tabuchi et al., 2002, 2010), characterized by peculiar sorting and recycling kinetics into endolysosomal compartment (White et al., 2016; Xu et al., 2017). In fact, (+)IRE DMT1 has prevalent surface expression, it is internalized from the plasma membrane without efficient recycling and targeted to lysosomes, while (–) IRE DMT1 is efficiently sorted to recycling endosomes upon internalization (Lam-Yuk-Tseung and Gros, 2006). Moreover, it was shown that expression of (–) IRE DMT1, increased by L-DOPA, could have a role in the development of L-DOPA toxicity, significantly counteracted by (–) IRE DMT1 silencing in primary cortical neurons (Du et al., 2009). Again, iron and DMT1 are involved in the pathogenesis of neurodegenerations with multifactorial origin, such as idiopathic PD, as shown by Salazar et al. (2008). They reported an increase of iron and DMT1 levels in the substantia nigra of post-mortem brain of PD patients and in the ventral mesencephalon of the PD mouse model of 1-methyl-4-phenyl-1,2,3,6-tetrahydropyridine (MPTP) intoxication. Furthermore, the authors importantly showed that the mk/mk microcytic mice and the Belgrade rat model, both harboring a DMT1 point mutation G185R with consequent impairment of iron uptake (Fleming et al., 1997, 1998), were protected from neurotoxicity induced by MPTP or by 6-hydroxidopamine (6-OHDA), respectively, thus highlighting an iron- and DMT1-dependence of the toxins mechanism of action. Again, in MES23.5 dopaminergic neurons and in the substantia nigra of the 6-OHDA lesioned rats, as a PD model, iron accumulation was associated to NMDA receptors activation, through the up-regulation of (+)IRE DMT1 and down-regulation of the iron exporter ferroportin (FPN1) (Xu et al., 2018). In this model, iron increase was counteracted by the non-competitive NMDA antagonist MK-801 and by the selective NMDA antagonist (2*R*)-amino-5-phosphonovaleric acid (AP5). Accordingly, iron and DMT1 protein accumulation were also found in the substantia nigra of the NF-κB/c-Rel knockout mice, a model of neurodegeneration with Parkinsonism (Baiguera et al., 2012). Ferrous iron and DMT1 up-regulation are also linked to neuroinflammatory signaling pathways, downstream to the NF-κB/RelA Lys310 acetylation-mediated cell death, during the early phase of brain ischemia, as demonstrated in the well-studied models of i*n vivo* mouse brain ischemic neurodegeneration, subjected to transient middle cerebral artery occlusion tMCAO, and in mouse primary cortical neurons subjected to oxygen-glucose-deprivation (OGD) (Ingrassia et al., 2012). Moreover, (–) IRE DMT1 was shown to contribute to increased ferrous iron uptake subsequent to treatment with 1-methyl-4-phenylpyridinium, MPP (+), in the MES23.5 model of dopaminergic neurons, fully antagonized by iron chelation with Desferal (Zhang et al., 2009), known to prevent dopaminergic neuronal death in MPTP-treated mice (Guo et al., 2016). Incidentally, the conditional overexpression of H-ferritin in TH positive neurons of the substantia nigra counteracted the increased level of iron and DMT1 in the transgenic mice, upon treatment with the proteasome inhibitor lactacystin, thus providing a genetic model of iron chelation (Zhu et al., 2010). In this regard, not only the epigenetic modulation but also the post-translational proteasomal degradation of DMT1 plays a role in DMT1-associated neurodegeneration. In fact, the E3-ubiquitin ligase Parkin, when overexpressed in SH-SY5Y neuroblastoma cells, increased proteasomal degradation of 1B DMT1 for both (+) IRE and (–) IRE isoforms (Roth et al., 2010). The authors also showed an accumulation of both (+) IRE and (–) IRE DMT1 isoforms in human lymphocytes of early onset patients with familial PD, bearing homozygous deletion of Parkin exon 4, and in the mouse brain of the E3 ubiquitin ligase Parkin knockout. Incidentally, another form of familial PD was identified with a mutation in the gene coding for the Vacuolar Protein Sorting 35, VPS35 (Zavodszky et al., 2014), which is responsible for the correct endosomal recycling and trafficking of DMT1 (Tabuchi et al., 2010), underpinning the derangement of DMT1 in autophagosomal localization as a basal mechanism involved in the disease.

The recent development of a transgenic mouse model of DMT1 overexpression has highlighted the relevant point that DMT1 overexpression alone is not enough to cause neurodegeneration (Zhang et al., 2017). In fact, DMT1 transgenic mice showed selective iron accumulation in the substantia nigra at 18 months of age, only when fed with an iron-supplemented diet. DMT1 expressing mice also showed increased levels of Parkin, as a compensative neuroprotection mechanism. However, DMT1 overexpression in the double mutant mice with Parkin null background, generated to avoid Parkin neuroprotective effect, showed no vulnerability against dietary iron intake, while exposure to 6-hydroxydopamine led to increased neurotoxicity.

Interestingly, with respect to brain iron compartmentalization in PD, post-mortem brain sections of PD patients showed a peculiar redistribution, with decreased levels of iron and iron transporters in the temporal cortex, if compared with the substantia nigra (Yu et al., 2013). Furthermore, several reports have recently highlighted that, during aging, iron and DMT1 accumulate in the frontal cortex of the APP_SWE_/PS1_ΔE9_ transgenic mouse model (Xian-hui et al., 2015), where both (–)/(+) IRE DMT1 were also increased in the cortex and hippocampus compared with wild type-control (Zheng et al., 2009), potentially contributing to increasing to the risk of developing AD. Interestingly, (–)/(+) IRE DMT1 up-regulation in the cortex and hippocampus of the APP_SWE_/PS1_ΔE9_ transgenic mouse model was recently shown as a consequence of down-regulation of Ndfip1, the NEDD4 family interacting protein 1 (Tian et al., 2018), which modulates DMT1 degradation through ubiquitination pathway (Foot et al., 2011). Again, (–)IRE DMT1 up-regulation was associated with a quantitative, age-dependent increase of heavy metals in the frontal cortex of a ”natural” rodent model of AD, named Octodon degus (Braidy et al., 2017), with impaired lysosomal function. Interestingly, in support for the presence of common mechanisms involving iron metabolism in different neurodegenerative diseases, the knockout mice lacking the ferroxidase Ceruloplasmin at 6 months of age showed Parkinsonian neurodegeneration with nigral iron accumulation and neuronal loss, which was prevented by iron chelation (Ayton et al., 2014). However, more recently, it was reported that Alzheimer models were induced in the knockout mouse of Ceruloplasmin either by injection of A$\text{β}{}_{25-35}$ into the lateral ventricle of the brain or by transgenic APP expression, with a consequential increase in memory impairment and hippocampal iron accumulation mediated by (–) IRE DMT1 and changes in ROS levels (Zhao et al., 2018).

In conclusion, these findings highlight the role of DMT1 in the pathogenesis of several, multifactorial and genetic neurodegenerative diseases induced by known neurotoxic stimuli.

## Neurodegeneration With Brain Iron Accumulation (NBIA)

Neurodegeneration with Brain Iron Accumulation (NBIA) encompasses a heterogeneous group of rare disorders characterized by abnormal progressive iron accumulation in specific brain areas. Their prevalence is estimated between 1 and three individuals in a million (Gregory and Hayflick, 2013). Clinical signs include dystonia, tremor, bradykinesia, rigidity, postural instability, optic atrophy or retinal degeneration, neuropsychiatric abnormalities ranging from rapid neurodevelopmental regression in infancy to minor cognitive impairment in adulthood (Gregory and Hayflick, 2013). To date, 12 disease-associated genes leading to NBIA have been identified; however, around 20% of cases are still genetically undefined (Arber et al., 2016; Levi and Tiranti, 2019). Only two genes, namely FTL (ferritin light chain) and CP (ceruloplasmin), are directly associated with iron homeostasis (Levi and Rovida, 2015; Kassubek et al., 2017). While the other ten genes encode proteins either with various functions in lipid metabolism, lysosomal activity and autophagic processes or with still unknown mechanisms: PANK2 (Pantothenate kinase 2), COASY (Coenzyme A synthase), PLA2G6 (Phospholipase A2), C19orf12 (Chromosome 19 open reading frame, with transcript of unknown function), FA2H (fatty acid 2-hydroxylase), ATP13A2 (ATPase cation transporting 13A2), WDR45 (WD repeat containing protein 45, beta-propeller protein), DCAF17 (DDB1 and CUL4-associated factor 17), SCP2 (sterol carrier protein 2) and GTPBP2 (GTP binding protein 2). Although demonstration of iron accumulation in the brain is essential for the diagnosis of NBIA, the impairment of iron metabolism in the different NBIA subclasses has not been clarified. Ingrassia et al. (2017) have recently shown an altered pattern of iron transporters with iron overload in patients’ fibroblasts with WDR45 mutations, a NBIA gene with a predicted role in autophagy (Hayflick et al., 2013). PLA2G6 knockout mice (Beck et al., 2015) and fibroblasts of a patient with Neuroferritinopathy (Barbeito et al., 2010) also showed altered expression of iron transporters with DMT1 up-regulation.

## DMT1 and Signaling Contributions to Neurodegeneration

Of abiding interest is the influence of inflammatory signaling pathways consequential upon heavy metal intoxication, which induces cytokine production and pro-inflammatory stimuli activation. Inflammation, concomitant to oxidative stress, is present in several neurodegenerative diseases. Noteworthy, the NF-κB signaling induces a transient rise of intracellular iron immediately after a short stimulation with Lipopolysaccharide (LPS), with consequential NF-κB nuclear activation already present at 15 min, as revealed in hepatic macrophages by electromobility shift assay, with a kB specific probe (Xiong et al., 2003). Intriguingly, later findings showed how LPS and NF-κB can regulate specific DMT1 isoforms, as later discussed, with apparently divergent responses at the post-translational level, due the activity of both NEDD4 family interacting protein 1 (Ndfip1) and E3 ubiquitin ligase Parkin. This critical regulatory system may represent a useful target in the prevention of metal accumulation, by DMT1 post-translational degradation, and neuronal cell death. In fact, overexpression of Ndfip1 was demonstrated to have a neuroprotective effect from metal toxicity with ubiquitination and degradation of DMT1 in human primary cortical neurons (Howitt et al., 2009) and after acute cortical brain injury (Sang et al., 2006). Accordingly, Ndfip1 knockout mice revealed a significant increase of intestinal DMT1 with higher serum iron levels, transferrin saturation and inflammation (Foot et al., 2011). Moreover, ferrous iron and LPS induced Ndfip1 protein expression in MES23.5 dopaminergic cell lines, were counteracted by iron chelation with deferoxamine (Xu et al., 2013). Xu and colleagues described Ndfip1 regulatory mechanism mediated by NF-κB activation, in agreement with previous results obtained with LPS stimulation (Xiong et al., 2003), demonstrating how BAY11-7802, an inhibitor of NF-κB activation, significantly decreased mRNA expression levels of Ndfip1 in response to both ferrous iron and LPS.

However, while Ndfip1-mediated post-translational regulation was mostly found associated to pan-DMT1 response, a parallel DMT1 isoform-specific, translational and post-translational, regulation has arisen. In fact, epigenetic studies contributed to elucidate the acetylome-dependent NF-κB regulation of 1B DMT1 promoter. Particularly, the acetylation of NF-κB/RelA at Lys310 activates the NF-κB-dependent response, a well-studied epigenetic regulation in post-ischemic injury (Lanzillotta et al., 2010), with consequent 1B/(–)IRE DMT1 up-regulation in models of differentiated human neuroblastoma and in mouse primary cortical neurons exposed to OGD, or transient middle cerebral artery occlusion (tMCAO) (Ingrassia et al., 2012), and also in undifferentiated P19 embryonic carcinoma cells (Paradkar and Roth, 2006a,Paradkar and Roth, 2006b). In this regard, the isoform-specific regulation of DMT1 could possibly explain the apparent discrepancy emerged between translational and post-translational regulation of Ndfip1. In fact, the Parkin-dependent post-translational degradation of 1B DMT1 isoform was shown to influence metal transport in SH-SY5Y, human neuroblastoma cells, overexpressing wild-type and mutant forms (Park-T240R) of human Parkin, while 1A DMT1 isoform resulted unaffected (Roth et al., 2010). Roth et al. (2010) importantly showed similar findings in human B lymphocyte cell lines derived from a PD patient carrying the homozygous deletion of Parkin exon 4, and in Parkin knockout mice where a clear (–) IRE DMT1 isoform specific degradation occurs, while the (+) IRE isoform expression is not altered. To this purpose, clear-cut data obtained from studies on HEK293 cells highlighted the isoform-dependence in DMT1 post-translational regulation (Garrick et al., 2012). Taking advantage of the peculiar expression level of 1A DMT1 isoform, which is high in HEK293 cell lines compared to neuronal cells, Garrick and colleagues clearly showed that in HEK293, 1A DMT1 is not a target for Parkin ubiqitination. In fact, transient transfection of Parkin in two HEK293F cell lines, with tetracycline-dependent overexpression of 1A/(+)IRE DMT1 and 1B/(–)IRE DMT1 isoforms respectively, allowed to detect a 1B isoform isoform-specificity of Parkin ubiquitination. In agreement with these findings, the Ndfip1 knockout mice showed a rise of intestinal pan-DMT1 (Foot et al., 2011), a district with predominant expression of 1A DMT1, further supporting the Parkin specificity for 1B/(–)IRE DMT1 isoform. Altogether, these results highlighted the heterogeneous post-translational regulation of DMT1 isoforms. On the other hand, data collected so far clearly claims the need for further studies in this regard. In fact, while Parkin induces DMT1 degradation in several models of PD, the loss of Ndfip1 was associated to DMT1 increase in ventral mesencephalic neuronal cultures of Ndfip1 knockout mice (Howitt et al., 2014). However, Howitt and colleagues concomitantly found increased Ndfip1 levels in the substantia nigra of PD patients, associated with the increase of (+) IRE DMT1. The authors suggested that this DMT1 up-regulation, parallel to Ndfip1 increase, could be accounted to the prevalence of translational regulation, respect to the post-translational one, with an increase of DMT1 isoforms due to iron exposure (Salazar et al., 2008), hypoxia (Lis et al., 2005), and NF-κB activation (Paradkar and Roth, 2006a,Paradkar and Roth, 2006b; Ingrassia et al., 2012).

In addition, signaling derangement with DMT1 misregulation was also presents in Alzheimer models. In fact, the decrease of Ndfip1 in the cortex and hippocampus of APP_SWE_/PS1_ΔE9_ transgenic mice was associated with the up-regulation of both (–) and (+) IRE DMT1 isoforms (Tian et al., 2018). Tian et al. (2018) also showed how the down-regulation of DMT1, with reduced iron uptake and decreased Aβ(1–42) peptide secretion, occurs after transient overexpression of Ndfip1 in SH-SY5Y human neuroblastoma cells stably over-expressing APP_sw_. Accordingly, in immortalized microglia cells, the Aβ (1–42) treatment induced pan-DMT1 up-regulation (McCarthy et al., 2018). Comprehensively, all these evidences lead to hypothesize a signaling mechanism accounting for the activation of specific ubiquitination pathways in the regulation of DMT1 isoforms.

In conclusion, the complex transcriptional and post-translational coordination of DMT1 isoforms may be underpinning to divalent metals exposure and neuroinflammation in PD, AD or any other trauma to the CNS. To this purpose, evident signs of gliosis and microglia activation (Toklu et al., 2018), or a mild inflammatory profile at pre-motor stage in the NF-κB/c-Rel^–/–^ mouse model of late-onset Parkinsonism, without gliosis at 18 months of age, were described (Porrini et al., 2017). Interestingly, the damage to the substantia nigra in PD induces TNF alpha and other cytokines that bind to their neuronal receptors with consequent NF-κB translocation to the nucleus, 1B DMT1 up-regulation and iron accumulation with cellular damage (Roth, 2009). In PD, iron and (+) IRE DMT1 are up-regulated in the dopaminergic, neuromelanin positive neurons of the substantia nigra in PD patients (Salazar et al., 2008). In accordance with the previous evidence, the early signs of neuroinflammation present in the NF-κB/c-Rel knockout mice, considered as a model of late-onset Parkinsonism (Baiguera et al., 2012), induced an increase of nigral iron and DMT1 gene expression in the striatum and mesencephalon. An analogous mechanism is involved during the early phase of brain ischemia, both *in vitro* and *in vivo* (Ingrassia et al., 2012). Accordingly, pretreatment with Tanshinone IIA, a phenanthrenequinone derivative, with anti-inflammatory potential against the NF-κB pathway, exerted neuroprotection against ischemic and hypoxic damage with down-regulation of DMT1 and TfR in cerebral ischemic rats subjected to *in vivo* tMCAO (Yang L. et al., 2011).

Furthermore, a close relationship between inflammation and ferrous iron increase, mediated by enhanced DMT1 gene expression, emerges in primary hippocampal astrocytes upon interferon gamma treatment (Pelizzoni et al., 2013). In addition, the inflammation-dependent increase of NTBI via up-regulated expression of pan-DMT1 is evident in immortalized mouse brain microglia cells, where LPS treatment up-regulates both DMT1 mRNA and protein levels, with concomitant extracellular acidification, due to metabolic changes. This leads to a maximum DMT1 uptake, and treatment with Amiloyd β (1-42), increases DMT1 mRNA and NTBI uptake (McCarthy et al., 2018). Moreover, inflammatory stimuli led to DMT1 up-regulation and ferroportin down-regulation, at both mRNA and protein levels in primary rat hippocampal neurons (Urrutia et al., 2013), in which a significant increase in cell death and oxidative stress was observed after treatment with Aβ-treated astrocyte- or microglia-conditioned media (Urrutia et al., 2017). These evidences show a complex panel about the contribution of the altered homeostasis of DMT1 regulation and iron accumulation, closely related to neuroinflammation, in several neurodegenerative disorders.

Noteworthy, iron overload induces cellular oxidative stress with increased lipid peroxidation, protein and nucleic acid modifications associated to neurodegenerative diseases (Liu et al., 2019). Importantly, under pathological conditions with inflammation, Nitric Oxide (NO) production is increased and aberrantly S-nitrosylated proteins can induce neurodegeneration, like the endogenous S-nitrosylation of DMT1 found in the substantia nigra of post-mortem PD brains (Liu et al., 2018). Interestingly, Liu et al. (2018) pointed the attention on the important aspect that, like extracellular acidification, the increased S-nitrosylation may enhance DMT1 uptake and cell death in the substantia nigra. Moreover, intra-nigral injection of LPS induced NO production and increased neuronal cell death, specifically blocked by the NO synthase inhibitor L-NAME or by Ebselen, a selective inhibitor of ferrous iron uptake by DMT1. Interestingly, lipids peroxidation also increased in the striatum of 100 week aged knockout mice for calcium-independent phospholipase A2 beta (iPLA2β), an enzyme that catalyzes the hydrolysis of membrane glycerophospholipids into free fatty acids and lysophospholipids, as a model of PLA2G6/NBIA (Beck et al., 2015).

Again, inflammation, through the altered expressions of DMT1, ferroportin and hepcidin, induces iron accumulation in the central nervous system (Urrutia et al., 2013; You et al., 2017; Ganz, 2018).

**FIGURE 2:**
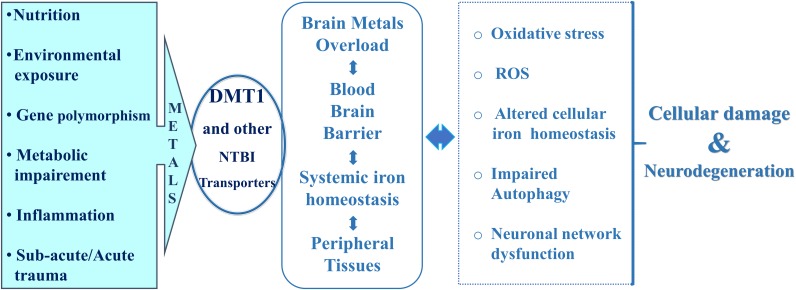
Cross-related events for DMT1-mediated cell death and neurodegeneration.

In this respect, hepcidin, the liver-derived peptide hormone, with iron-sensing competence through post-translational degradation of ferroportin, the only known iron-exporter so far identified, plays a remarkable role in the control of systemic iron homeostasis as its expression is up-regulated by inflammation (Ganz, 2013). Hepcidin significantly influences transferrin-bound-iron transport (Tf-Fe) and NTBI homeostasis with the involvement of the respective transporters (Du et al., 2015; Zhou et al., 2017). In addition, hepcidin deficiency has also been reported to be associated with iron overload in hereditary hemochromatosis (Papanikolaou et al., 2005). Recent evidences highlighted that hepcidin expression in the brain is low, according to Burkhart et al. (2016), and is upregulated in the central nervous system during pathophysiological inflammation, exerting a paracrine action on neurons through down-regulation of ferroportin expression (Urrutia et al., 2013; You et al., 2017; Vela, 2018). Expression of ferroportin was assessed in endothelial cells of the blood–brain barrier, in neurons, oligodendrocytes, astrocytes, in the choroid plexus and ependymal cells, as well as being identified in the synaptic vesicles fraction of purified rat brain synaptosomes (Wu et al., 2004). However, the iron exporter ferroportin is a hepcidin target in neurons, astrocytes and microglial cells (Urrutia et al., 2013; You et al., 2017). During inflammation, astrocytes drive iron influx from blood flow to the brain, with neuronal iron accumulation through microglia and astrocyte activity, resulting in neurocytotoxicity and neurodegeneration. Systemic hepcidin can cross the blood brain barrier, particularly when BBB is damaged by pathological conditions such as intracerebral hemorrhage and acute inflammation (Ostergaard et al., 2009; Xiong et al., 2016). It is noteworthy that hepcidin serum levels were significantly increased in PD patients after deep brain stimulation (Kwiatek-Majusuak et al., 2018) and in AD patients (Sternbreg et al., 2017). Therefore, the role of heavy metals absorption and accumulation in the central nervous system may represent a central event for several neurodegenerative diseases of both idiopathic and genetic origin, which arise from nutrition, lifestyle, environmental exposure, genetic polymorphisms, traumatic and neuroinflammatory events affecting the central nervous system (Figure 2).

## Author Contributions

RI, BG, and MM contributed to the writing of the manuscript. RI reviewed and edited the manuscript.

## Conflict of Interest Statement

The authors declare that the research was conducted in the absence of any commercial or financial relationships that could be construed as a potential conflict of interest.
